# The sirtuin 6 prevents angiotensin II-mediated myocardial fibrosis and injury by targeting AMPK-ACE2 signaling

**DOI:** 10.18632/oncotarget.20305

**Published:** 2017-08-17

**Authors:** Zhen-Zhou Zhang, Yu-Wen Cheng, Hai-Yan Jin, Qing Chang, Qian-Hui Shang, Ying-Le Xu, Lin-Xi Chen, Ran Xu, Bei Song, Jiu-Chang Zhong

**Affiliations:** ^1^ State Key Laboratory of Medical Genomics, Ruijin Hospital, Shanghai Jiao Tong University School of Medicine, Shanghai Key Laboratory of Hypertension, Shanghai Institute of Hypertension, Shanghai 200025, China; ^2^ Institute of Health Sciences, Shanghai Institute for Biological Sciences, Chinese Academy of Sciences, Shanghai 200025, China; ^3^ Department of Mental Health, Ruijin Hospital, Shanghai Jiao Tong University School of Medicine, Shanghai 200025, China; ^4^ Department of Cardiology and Institute of Clinical Medicine Research, Affiliated Hospital of Zunyi Medical College, Zunyi 563003, China; ^5^ Institute of Pharmacy and Pharmacology, University of South China, Hengyang 421001, China

**Keywords:** SIRT6, angiotensin-converting enzyme 2, connective tissue growth factor, fibrosis, myocardial injury

## Abstract

Sirtuin 6 (SIRT6) is an important modulator of cardiovascular functions in health and diseases. However, the exact role of SIRT6 in heart disease is poorly defined. We hypothesized that SIRT6 is a negative regulator of angiotensin II (Ang II)-mediated myocardial remodeling, fibrosis and injury. The male Sprague-Dawley rats were randomized to Ang II (200 ng/kg/min) infusion with an osmotic minipump and pretreated with recombinant plasmids adeno-associated viral vector (AAV)-SIRT6 (pAAV-SIRT6) or pAAV-GFP for 4 weeks. Ang II triggered downregulated levels of SIRT6 and angiotensin-converting enzyme 2 (ACE2) and upregulated expression of connective tissue growth factor (CTGF) and proinflammatory chemokine fractalkine (FKN), contributing to enhanced cardiac fibrosis and ultrastructural injury. Reduced levels of phosphorylated pAMPK-α, increased myocardial hypertrophy and impaired heart dysfunction were observed in both Ang II-induced hypertensive rats and ACE2 knockout rats, characterized with increases in heart weight and left ventricular (LV) posterior wall thickness and decreases in LV ejection fraction and LV fractional shortening. More importantly, pAAV-SIRT6 treatment strikingly potentiated cardiac levels of pAMPKα and ACE2 as well as decreased levels of CTGF, FKN, TGFβ1, collagen I and collagen III, resulting in alleviation of Ang II-induced pathological hypertrophy, myocardial fibrosis, cardiac dysfunction and ultrastructural injury in hypertensive rats. In conclusion, our findings confirmed cardioprotective effects of SIRT6 on pathological remodeling, fibrosis and myocardial injury through activation of AMPK-ACE2 signaling and suppression of CTGF-FKN pathway, indicating that SIRT6 functions as a partial agonist of ACE2 and targeting SIRT6 has potential therapeutic importance for cardiac fibrosis and heart disease.

## INTRODUCTION

Heart failure is growing epidemic with high morbidity and mortality at an international scale [1, 2]. The sirtuin SIRT6, a conserved nicotinamide adenine dinucleotide-dependent protein deacetylase, plays critical roles in the regulation of cardiovascular functions, energy metabolism and aging [3–5]. Both single- and double-SIRT6 knockout mice develop significant cardiac hypertrophy, fibrosis and dysfunction [6]. Myocardial samples from patients with heart failure showed markedly reduced SIRT6 levels, indicating an important role of the SIRT6 signaling in heart disease [6]. SIRT6 is highly expressed in heart and is an important modulator of cardiovascular functions in health and diseases [3, 5, 7].

Increasing evidence indicates the crosstalk between the SIRT signaling and the renin-angiotensin system (RAS) [6, 7]. Physiological, pharmacological and clinical studies have demonstrated that activation of the RAS is a key mediator of the progression of hypertension and heart failure [2, 8–9]. Angiotensin (Ang) II, a main biological peptide of RAS, plays an essential role in myocardial interstitial fibrosis by enhancing activation of connective tissue growth factor (CTGF)-fractalkine (FKN) signaling, contributing to increased myocardial hypertrophy, dysfunction and injury [10, 11]. ACE2 is considered as a potential therapeutic target of the RAS for the treatment of hypertension and heart failure and converts Ang II into Ang-(1–7), which, by virtue of its actions on the Mas receptor, opposes the molecular and cellular effects of Ang II [12–14]. As a critical enzyme in the metabolism of Ang II, ACE2 serves to directly balance the levels of Ang II and Ang (1–7). Absence of ACE2 triggers greater increases in cardiac CTGF-FKN signaling in ACE2-deficient mice in response to chronic Ang II infusion, leading to exacerbation of the Ang II-induced myocardial hypertrophy and ultrastructure injury [10]. Intriguingly, ACE2 expression is found to be regulated by SIRT1 and adenosine 5′-monophosphate-activated protein kinase (AMPK) activation results in increased ACE2 expression [15]. However, the interaction between the SIRT6 and AMPK/ACE2 signaling in heart disease is poorly defined. In the present study, we hypothesized that SIRT6 is a negative regulator of Ang II-mediated myocardial remodeling and fibrosis by targeting AMPK/ACE2 signaling. We evaluated the effects of Ang II and ACE2 deficiency on SIRT6 levels and regulatory roles of SIRT6 in AMPK/ACE2 signaling and Ang II-mediated heart disease by the use of ACE2-mutant and Ang II-infused hypertensive rats.

## RESULTS

### Downregulation of SIRT6 and ACE2 levels and enhanced myocardial hypertrophy are obversed in Ang II-induced hypertensive rats

**Figure 1:**
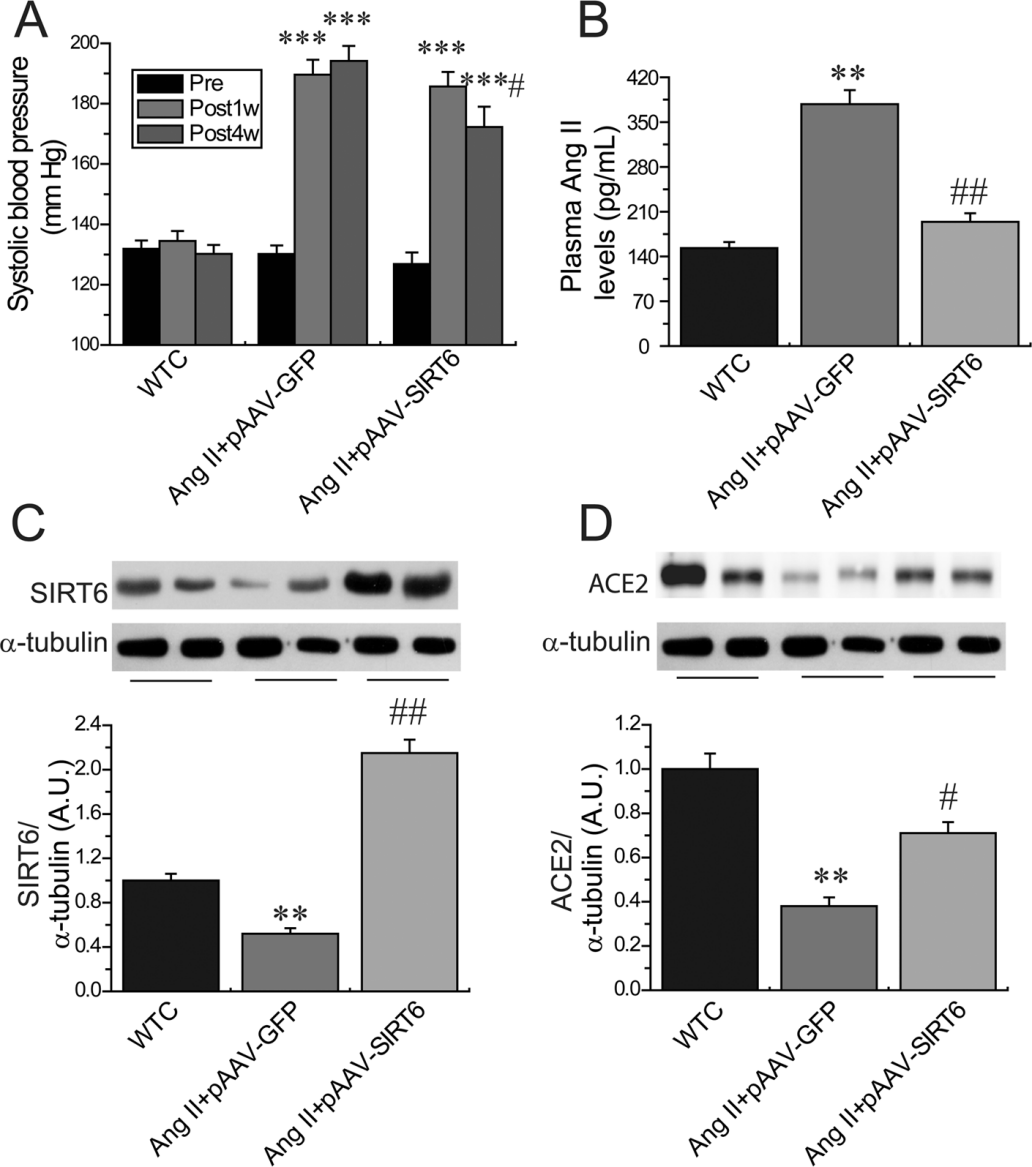
Systolic blood pressure levels and myocardial levels of SIRT6 and ACE2 in Ang II-infused rats (**A**) Systolic blood pressure levels were upregulated in hypertensive rats at 1 week and 4 weeks after Ang II infusion while reduced in pAAV-SIRT6-treated hypertensive rats at 4 weeks after Ang II infusion. (**B**–**D**), Plasma Ang II levels (B) and representative Western blotting (C, D) exhibiting that Ang II levels were enhanced and myocardial SIRT6 and ACE2 levels were declined in pAAV-GFP-treated hypertensive rats, which were prevented in pAAV-SIRT6-treated hypertensive rats. Pre = prior to treatment; Post 1w, after 1 week treatment; Post 4w, after 4 week treatment; WTC=wildtype control. *n* = 7–10 except for C and D where n=4. ^**^P < 0.01, ^***^P < 0.001 compared with WTC group; ^#^*P* < 0.05; ^##^*P* < 0.01 compared with Ang II+pAAV-GFP group.

**Figure 2:**
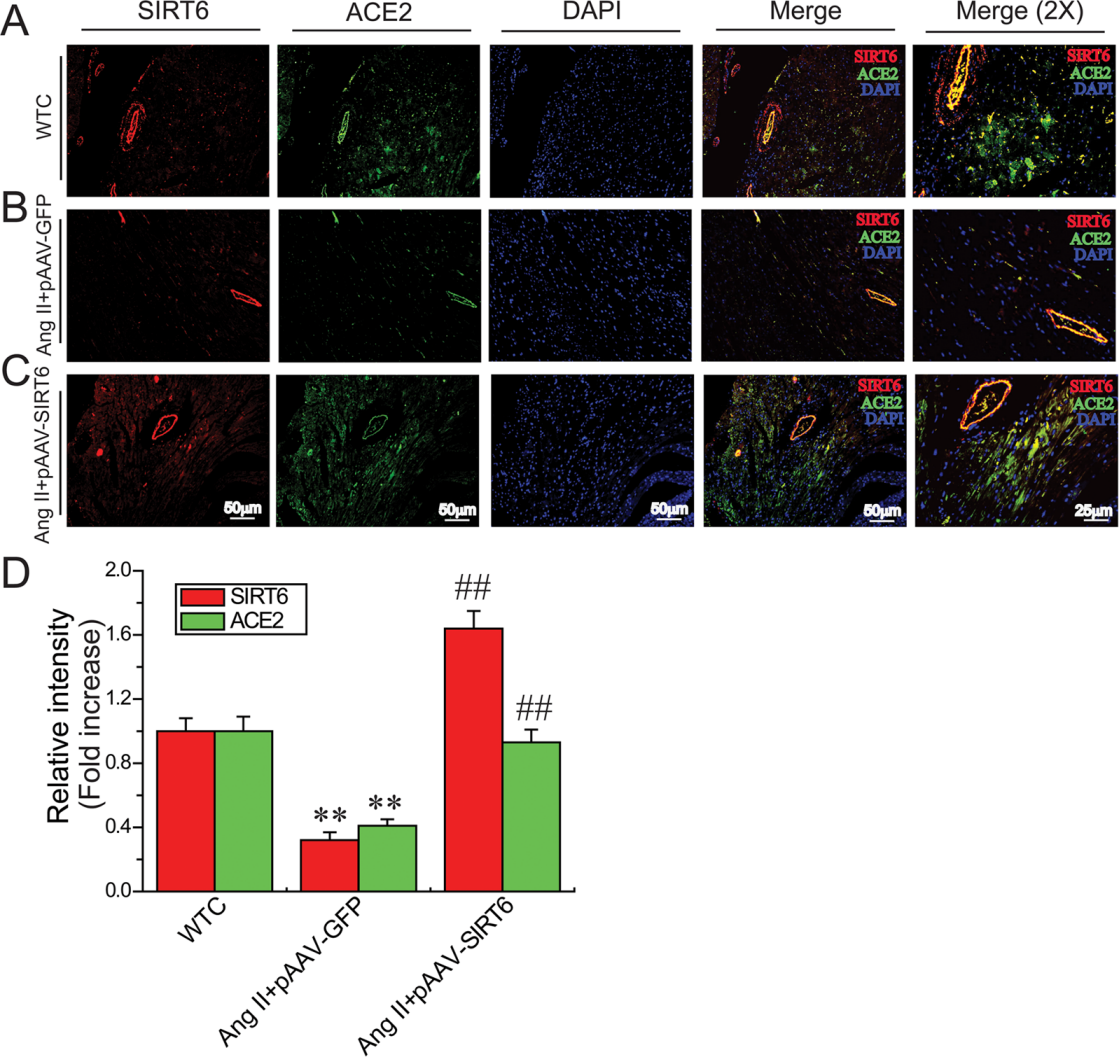
Myocardial SIRT6 and ACE2 levels in rat hearts Double immunofluorescence staining (**A**–**C**) and relative fluorescence values (**D**) illustrating myocardial levels of SIRT6 and ACE2 in rats. In immunofluorescence images, the red color represents SIRT6, the green color represents ACE2, and blue color represents DAPI stained nuclei. WTC=wildtype control; DAPI, 4′,6-diamidino-2-phenylindole. *n* = 4. ^**^P < 0.01 compared with WTC group; ^##^*P* < 0.01 compared with Ang II+pAAV-GFP group.

**Figure 3:**
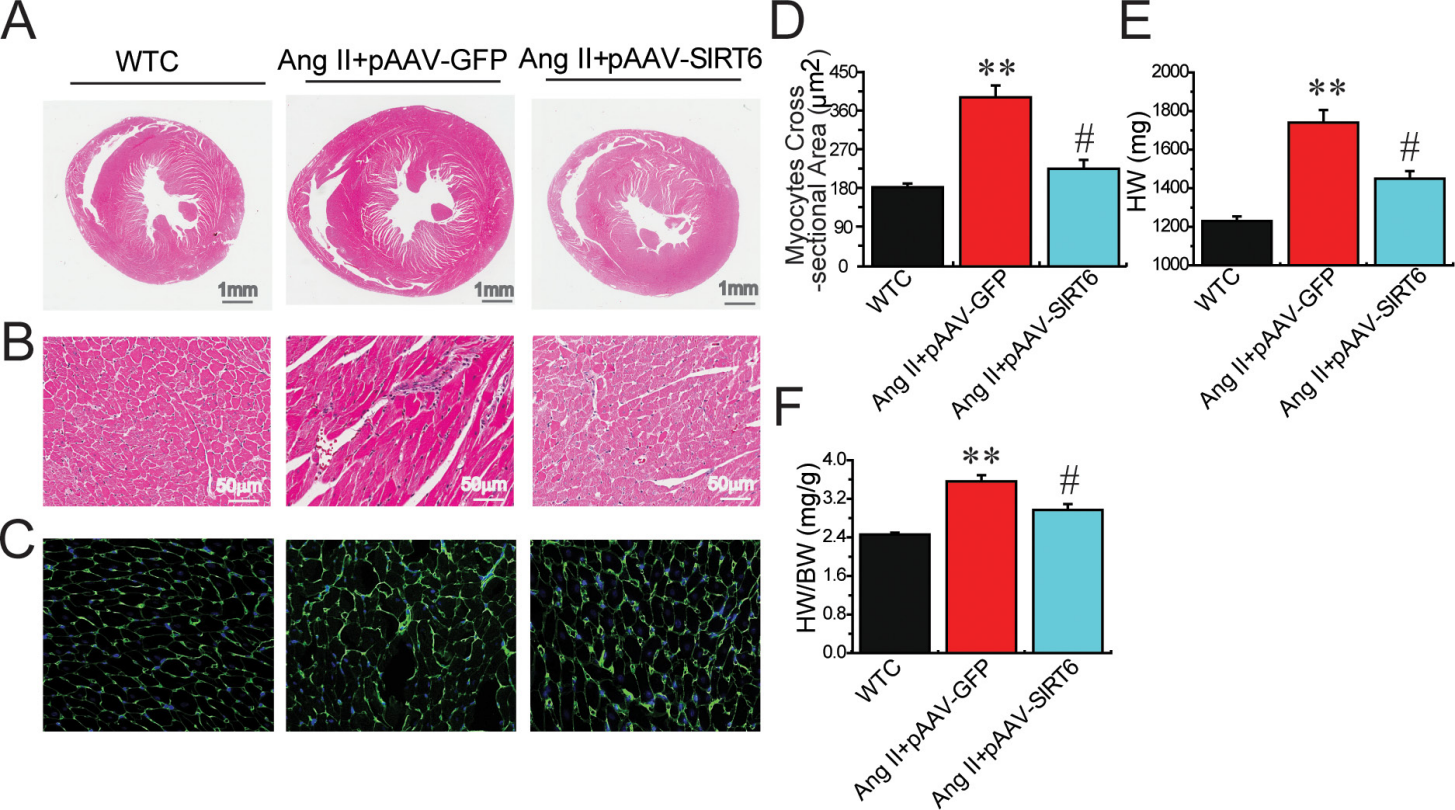
Effects of pAAV-SIRT6 treatment on myocardial hypertrophy in response to Ang II Representative hematoxylin and eosin (**A**–**B**) and Wheat Germ Agglutinin (WGA) staining (**C**–**D**) exhibiting attenuated myocardial hypertrophy in pAAV-SIRT6-treated hypertensive rats with decreases in HW (**E**) and the HW/BW ratio (**F**). WTC=wildtype control; HW, heart weight; BW, body weight. *n* = 7–10 except for D where *n* = 4. ^**^P < 0.01 compared with WTC group; #, *P*<0.05 compared with Ang II+pAAV-GFP group.

We firstly evaluated the effects of Ang II infusion on cardiac SIRT6 and ACE2 expression and myocardial hypertrophy. Ang II infusion led to marked increases in systolic blood pressure levels (Figure 1A) and downregulation of myocardial SIRT6 levels (Figure 1C) and ACE2 (Figure 1D) in the recombinant plasmids adeno-associated viral vector (AAV)-GFP (pAAV-GFP)-treated hypertensive rats associated with increased levels of plasma Ang II (Figure 1B). Double-immunofluorescence staining analysis further confirmed downregulated levels of SIRT6 and ACE2 (Figures 2B, 2D) in Ang II-mediated hypertensive rats when compared with wildtype (WT) control rats. In addition, histomorphometric analysis (Figures 3A, 3B) and Wheat germ agglutinin (WGA) staining (Figures 3C, 3D) demonstrated enhanced myocardial hypertrophy in Ang II-mediated hypertensive rats along with obvious increases in heart weight (HW; Figure 3E) and the ratio of HW and body weight (BW) (Figure 3F).

### ACE2-deficient rat developes downregulation of SIRT6 and p-AMPKα levels, increased myocardial fibrosis and impaired heart function

**Figure 4:**
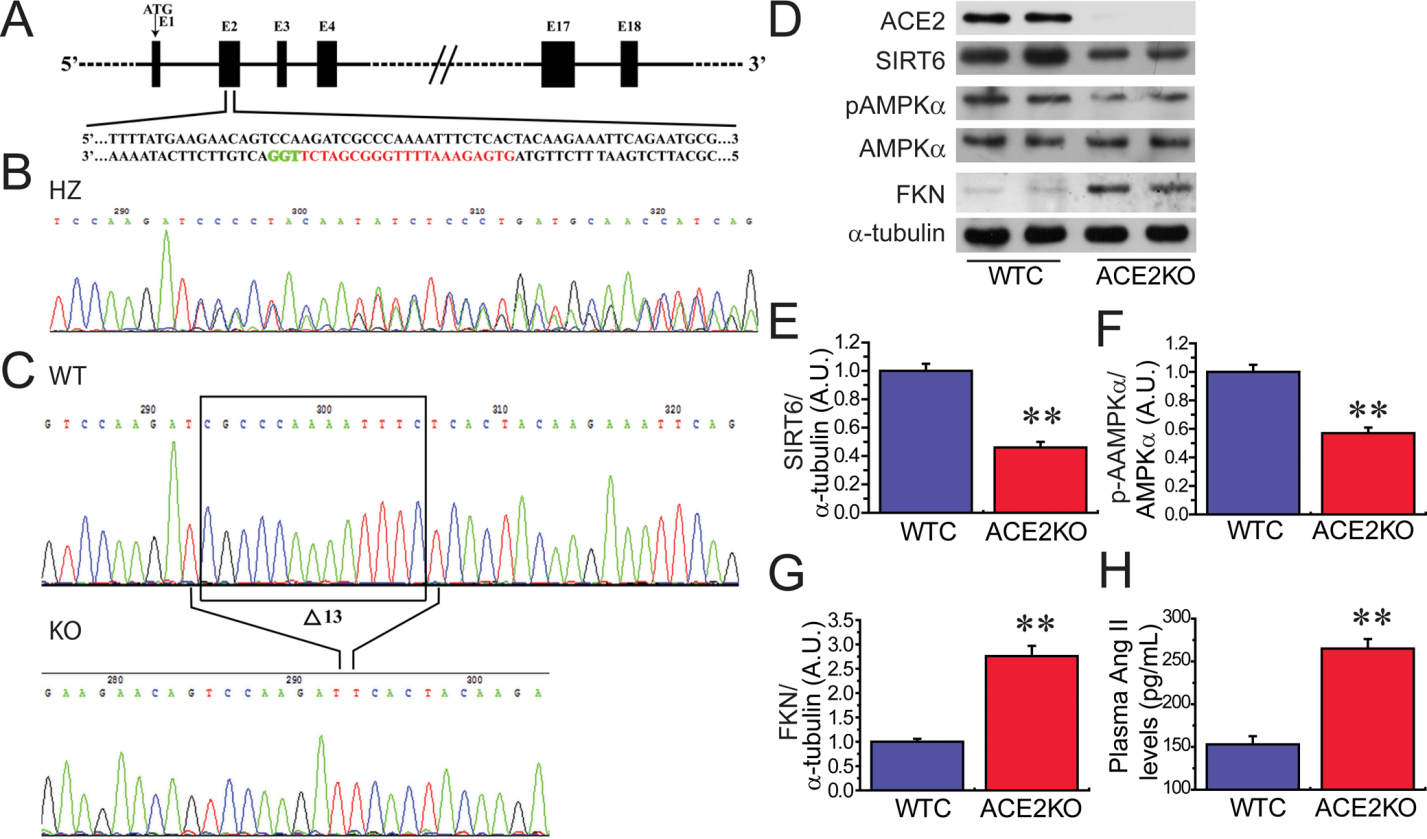
ACE2 deficiency results in decreases in cardiac SIRT6 and p-AMPKa levels and increased FKN expression (**A**) Schematic of the Sprague-Dawley (SD) rat ACE2 gene (E2). The translation start site (ATG) is located in E1, the transcription activator-like effector nuclease (TALEN) target sites are underlined, and the SphI restriction site in the spacer is highlighted in red. (**B**) Representative results from the DNA sequencing of founders. The sequencing chromatogram of heterozygous mutants revealed an indel that resulted in double peak traces. (**C**) DNA sequence chromatograms of polymerase chain reaction (PCR) products from wild-type (WT) and knockout (KO) rats illustrating the 13 bp deletion in the homozygous sample. D-H, Loss of ACE2 leads to reduced levels of SIRT6 (**D**, **E**) and pAMPKα (**F**) as well as elevated levels of cardiac FKN (**G**) and plasma Ang II (**H**) in ACE2KO hearts. WTC, wildtype control; KO, knockout; AMPK, adenosine 5′-monophosphate-activated protein kinase; ACE2, angiotensin-converting enzyme 2; FKN, fractalkine. *n* = 8–10. ^**^P < 0.01 compared with WTC group.

**Figure 5:**
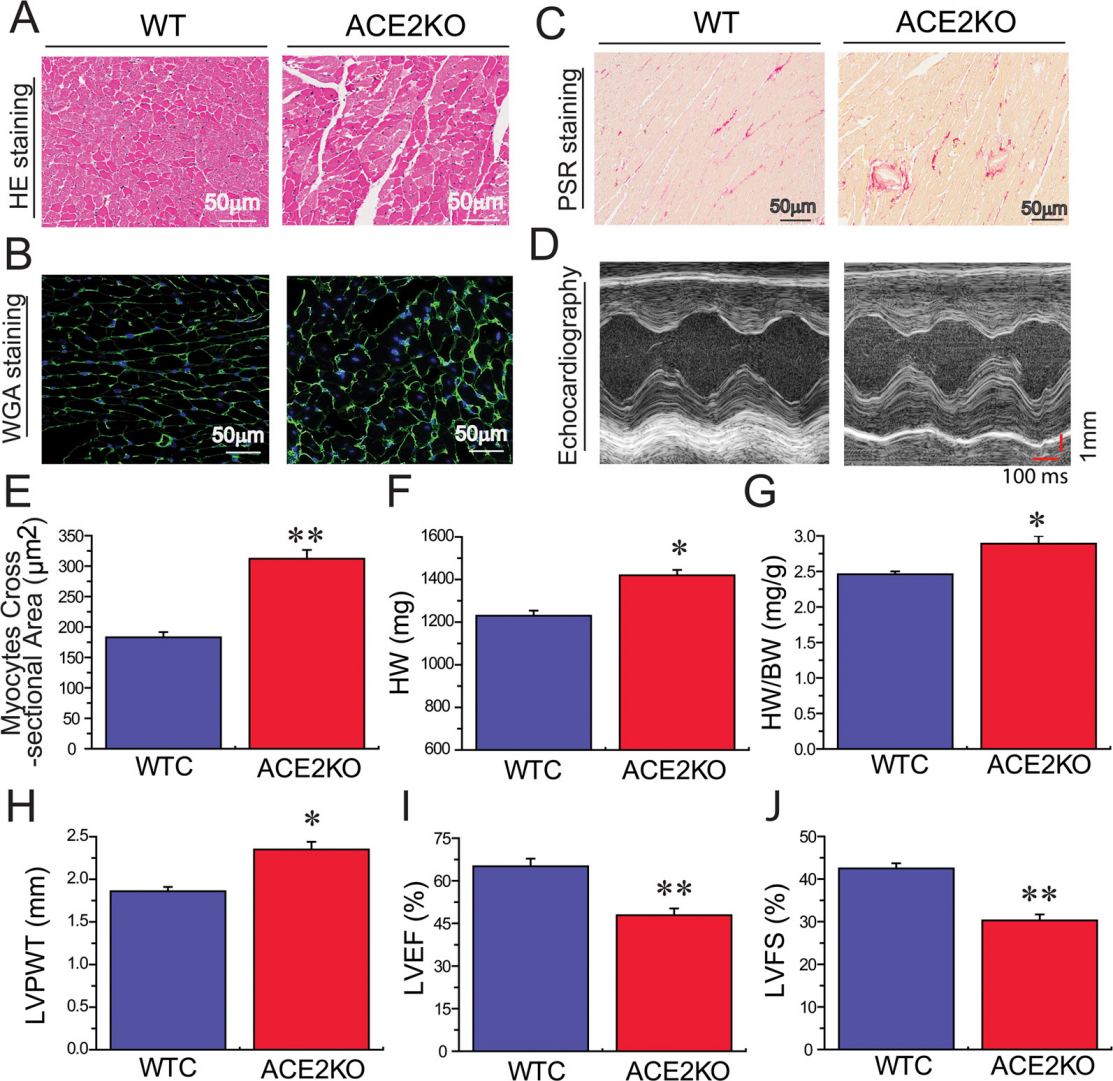
ACE2 deficiency results in increased cardiac hypertrophy, fibrosis and impaired heart function HE (**A**), WGA (**B**, **E**) and PSR (**C**) staining and echocardiography assessment (**D**) exhibiting greater cardiac hypertrophy and increased myocardial fibrosis in ACE2KO rats, along with marked increases in HW (**F**), HW/BW ratio (**G**) and LVPWT (**H**) and decreases in LVEF (**I**) and LVFS (**J**). In WGA images, the green color represents WGA, and blue color represents DAPI stained nuclei. KO, knockout; HE, Hematoxylin and eosin; PSR, Picrosirius red; WTC, wildtype control; HW, heart weight; BW, body weight; WGA, Wheat germ agglutinin; FKN, fractalkine; LVPWT, left ventricular (LV) posterior wall thickness; LVEF, LV ejection fraction; LVFS, LV fractional shortening. *n* = 8–10. ^**^P < 0.01 compared with WTC group.

We next evaluated the regulatory roles of ACE2 deficiency in levels of SIRT6 and phosphorylated AMPKα (pAMPKα) and pathological hypertrophy, fibrosis and cardiac function using ACE2 knockout (KO) rats. Loss of ACE2 resulted in reduced levels of SIRT6 (Figures 4D, 4E) and pAMPKα (Figure 4F) as well as marked increases in FKN expression (Figure 4G) and plasma Ang II levels (Figure 4H) in ACE2KO hearts. Histomorphometric analysis (Figure 5A), WGA (Figure 5B, 5E) and picrosirius red staining (Figure 5C) and echocardio-graphic assessment (Figure 5D) revealed greater cardiac hypertrophy, increased myocardial fibrosis and impaired systolic function in ACE2-deficient rats characterized with decreases in left ventricular (LV) ejection fraction (LVEF; Figure 5I) and LV fractional shortening (LVFS; Figure 5J). These changes were associated with marked increases in HW (Figure 5F), HW/BW ratio (Figure 5G) and LV posterior wall thickness (LVPWT; Figure 5H) in ACE2-null hearts.

### SIRT6 treatment prevented Ang II-induced myocardial hypertrophy and dysfunction by activation of AMPK/ACE2 signaling

**Figure 6:**
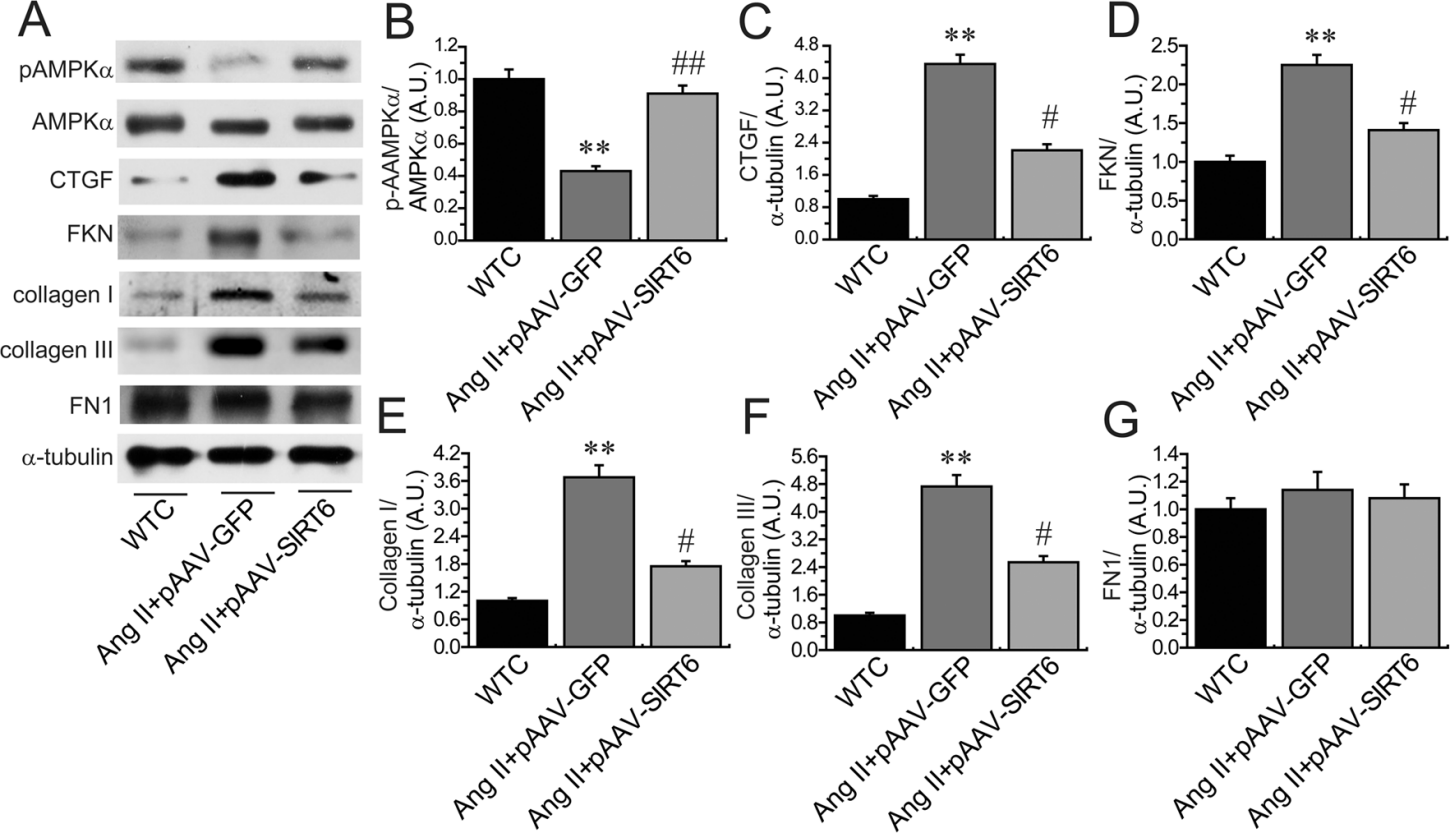
Cardiac levels of pAMPKa, CTGF and FKN in rat hearts The levels of pAMPKα (**A**–**B**), CTGF (**C**), FKN (**D**), collagen I (**E**), collagen III (**F**) and FN1 (**G**) were measured in rats by Western blotting analysis. WTC= wildtype control; AMPK, adenosine 5′-monophosphate-activated protein kinase; CTGF, connective tissue growth factor; FKN, fractalkine; FN1, fibronectin 1. n=5 for each group. ^**^P < 0.01, compared with WTC group; ^#^*P* < 0.05; ^##^*P* < 0.01 compared with Ang II+pAAV-GFP group.

**Figure 7:**
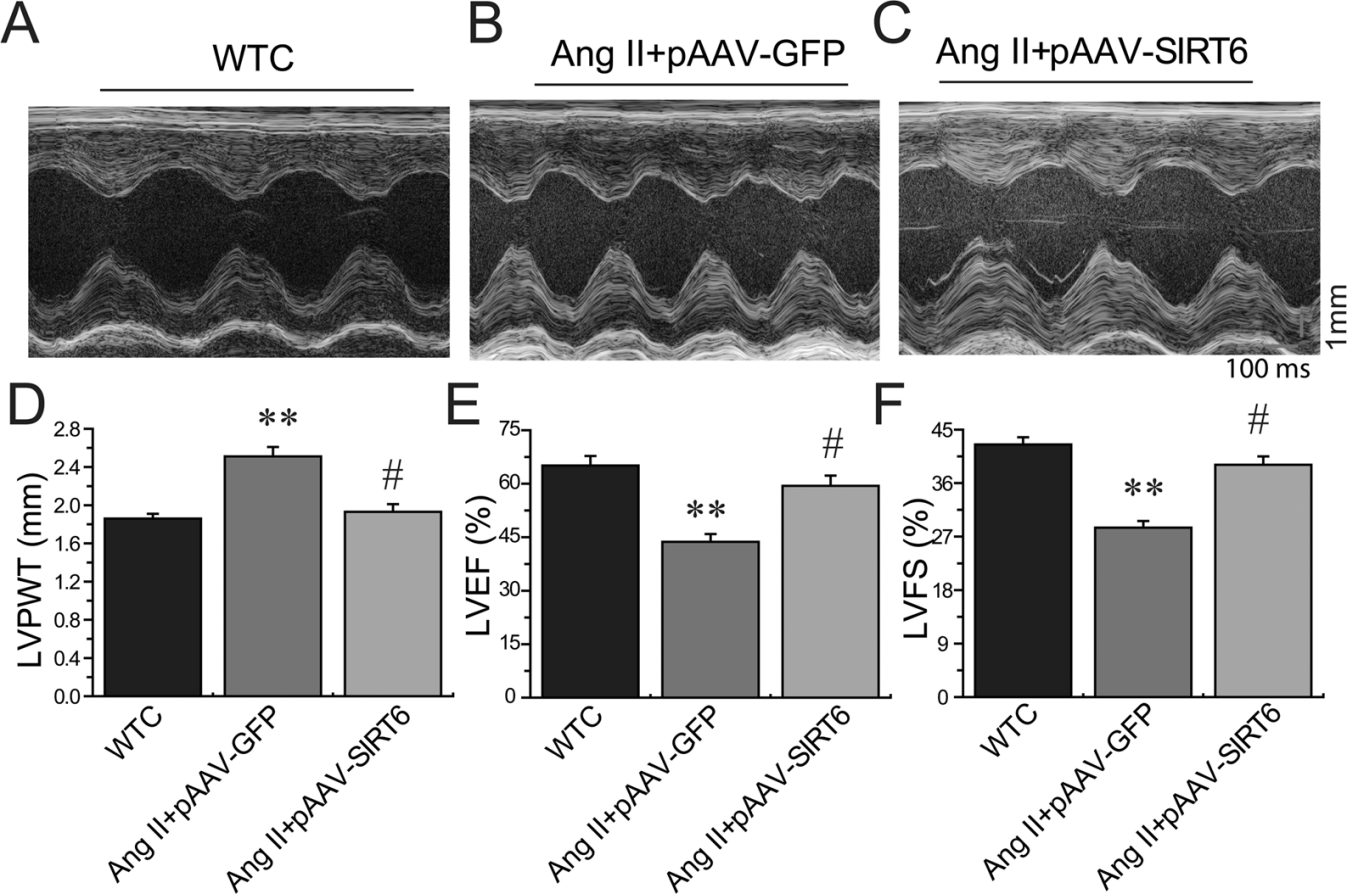
Effects of pAAV-SIRT6 treatment on systolic dysfunction in response to Ang II Echocardiographic assessment (**A**–**C**) showing decreased LVPWT (**D**) and improved systolic dysfunction in pAAV-SIRT6-treated hypertensive rats with increased levels of LVEF (**E**) and LVFS (**F**). WTC=wildtype control; LVPWT, LV posterior wall thickness; LVFS, LV fractional shortening; LVEF, LV ejection fraction. *n* = 5 for each group. ^**^P < 0.01, compared with WTC group; ^#^*P* < 0.05; compared with Ang II+pAAV-GFP group.

Compared with pAAV-GFP-treated hypertensive rats, administration of pAAV-SIRT6 significantly prevented Ang II-mediated pressor response at post 4-week treatment (Figure 1A) and myocardial hypertrophy (Figure 7C) in hypertensive rats with upregulated cardiac levels of ACE2 (Figure 1D) and pAMPKα (Figures 6A, 6B). These changes were associated with marked decreases in HW (Figure 3E), HW/BW ratio (Figure 3G) and LVPWT (Figure 7D) and augmentation of LVEF (Figure 7E) and LVFS (Figure 7F) in pAAV-SIRT6-treated hypertensive hearts. However, administration of pAAV-SIRT6 did not affect systolic blood pressure levels at post 1-week treatment (Figure 1A). Our data clearly demonstrate cardioprotective roles of SIRT6 in controlling cardiac remodeling and dysfunction via activation of the AMPK/ACE2 signaling.

### SIRT6 treatment prevented Ang II-induced myocardial fibrosis and injury by activation of AMPK-ACE2 pathway and suppression of CTGF-FKN signaling

**Figure 8:**
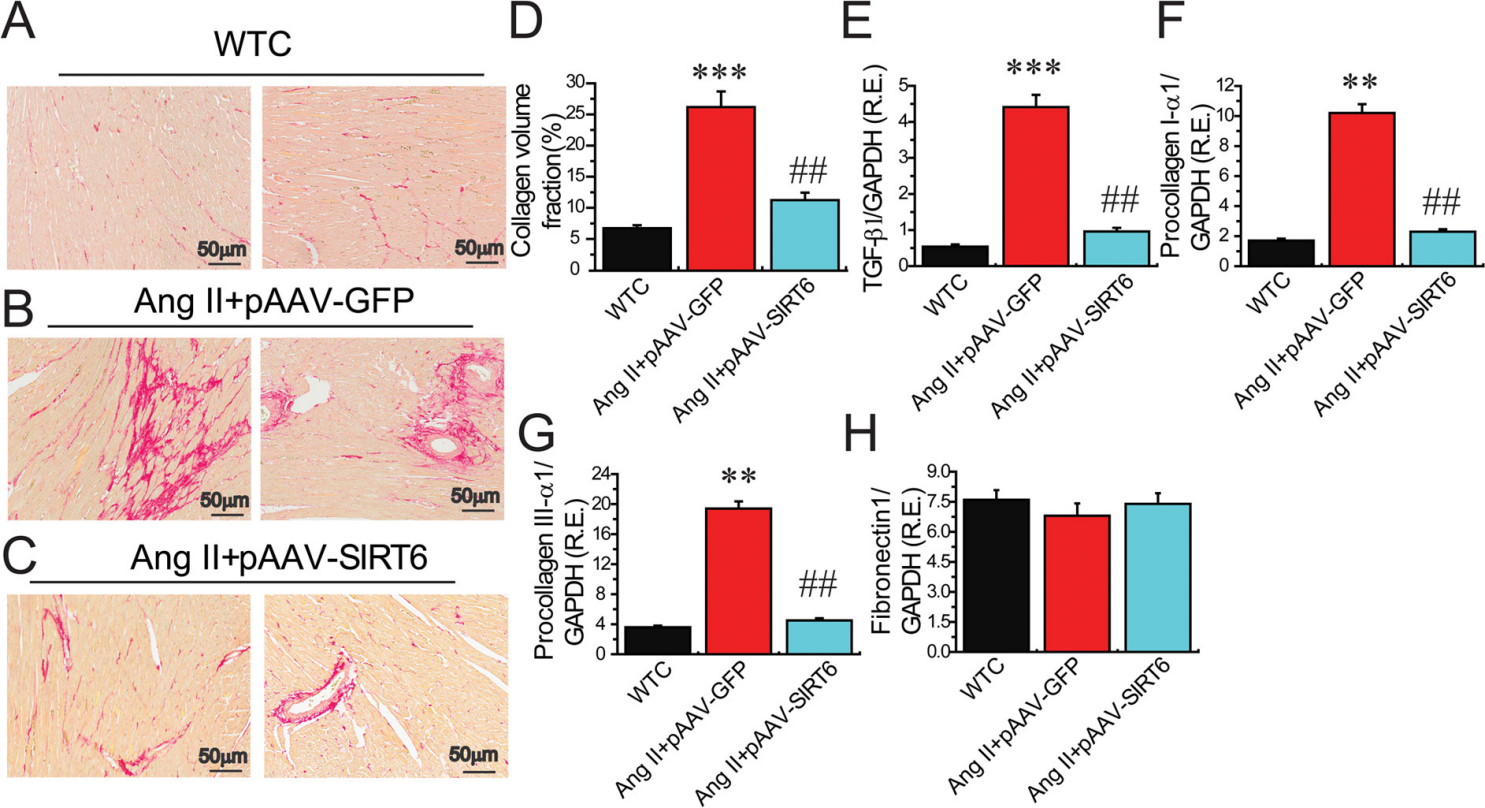
Effects of pAAV-SIRT6 treatment on myocardial fibrosis in response to Ang II (**A**–**D**) Representative images of Picrosirius red (PSR) staining (A–C) and collagen volume fraction (D) showed attenuated myocardial fibrosis in pAAV-SIRT6-treated hypertensive rats (′100 magnification). *n* = 4. (**E**–**H**) The real-time PCR analysis revealed the mRNA levels of fibrosis-related genes TGF-β1 (E), procollagen I-α1 (F), procollagen III-α1 (G), and fibronectin1 (H) in rat hearts. *n* = 6 for each group. GAPDH was used as an endogenous control. R.E.= relative expression, TGFβ1, transforming growth factor-β1. ^**^P < 0.01, ^***^P < 0.001 compared with WT control (WTC) group; ^##^*P* < 0.01 compared with Ang II+pAAV-GFP group.

**Figure 9:**
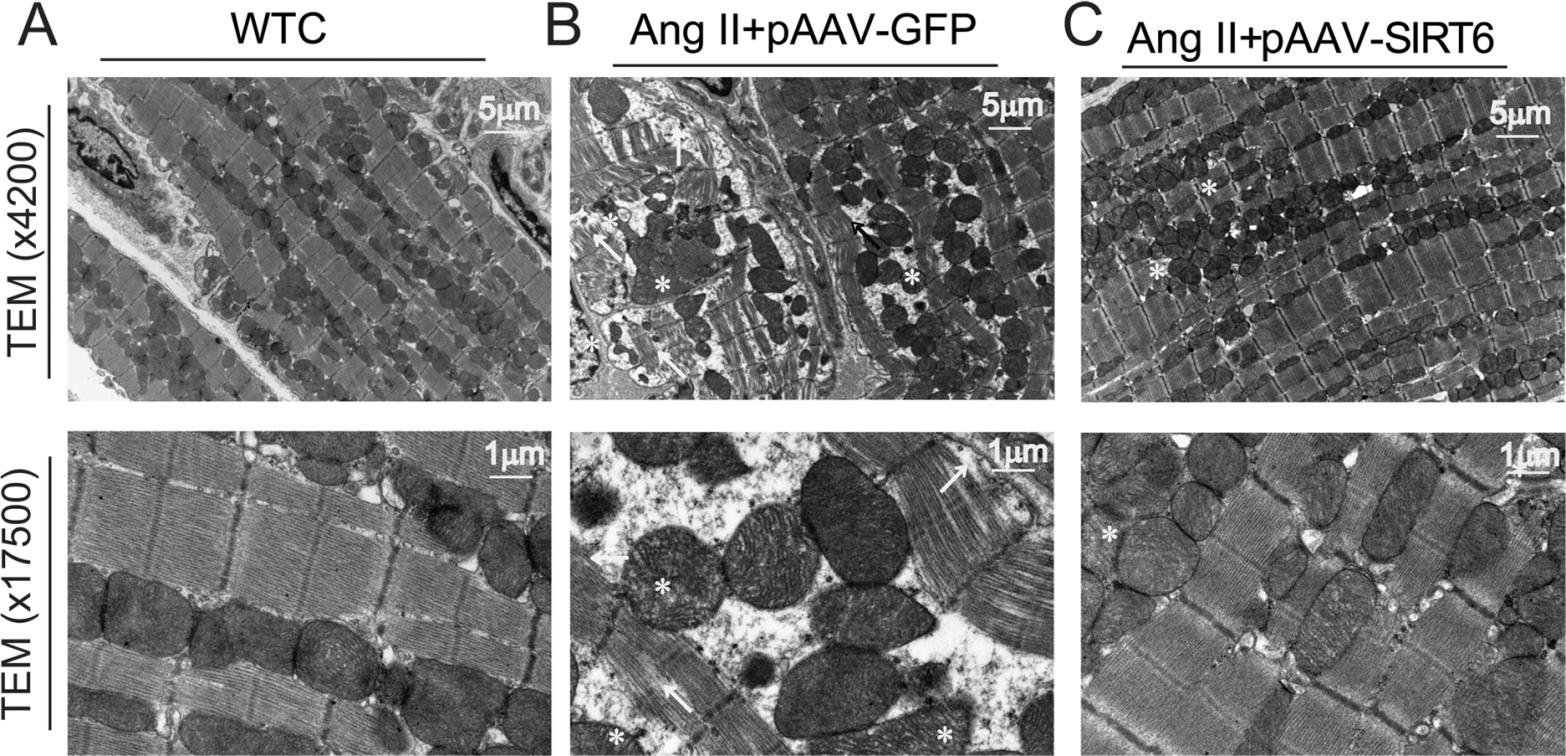
Effects of pAAV-SIRT6 treatment on myocardial ultrastructure injury mediated by Ang II The myocardial ultrastructural changes were observed in WT control (**A**), pAAV-GFP- (**B**) and pAAV-SIRT6-treated hypertensive rats (**C**) by transmission electron microscope analysis with×4200 and ×17500 magnification. Compared with WT control rats, severe myocardial ultrastructure injury was observed in pAAV-GFP-treated hypertensive rats, characterized with disruption or dissolution of myocardial myofilaments, myofilaments arranged irregularly and loosely (arrow), and vacuolar degenerational and swollen mitochondrias (star). Notably, myocardial ultrastructure injury were alleviated in hypertensive rats in response to pAAV-SIRT6 treatment.

**Figure 10:**
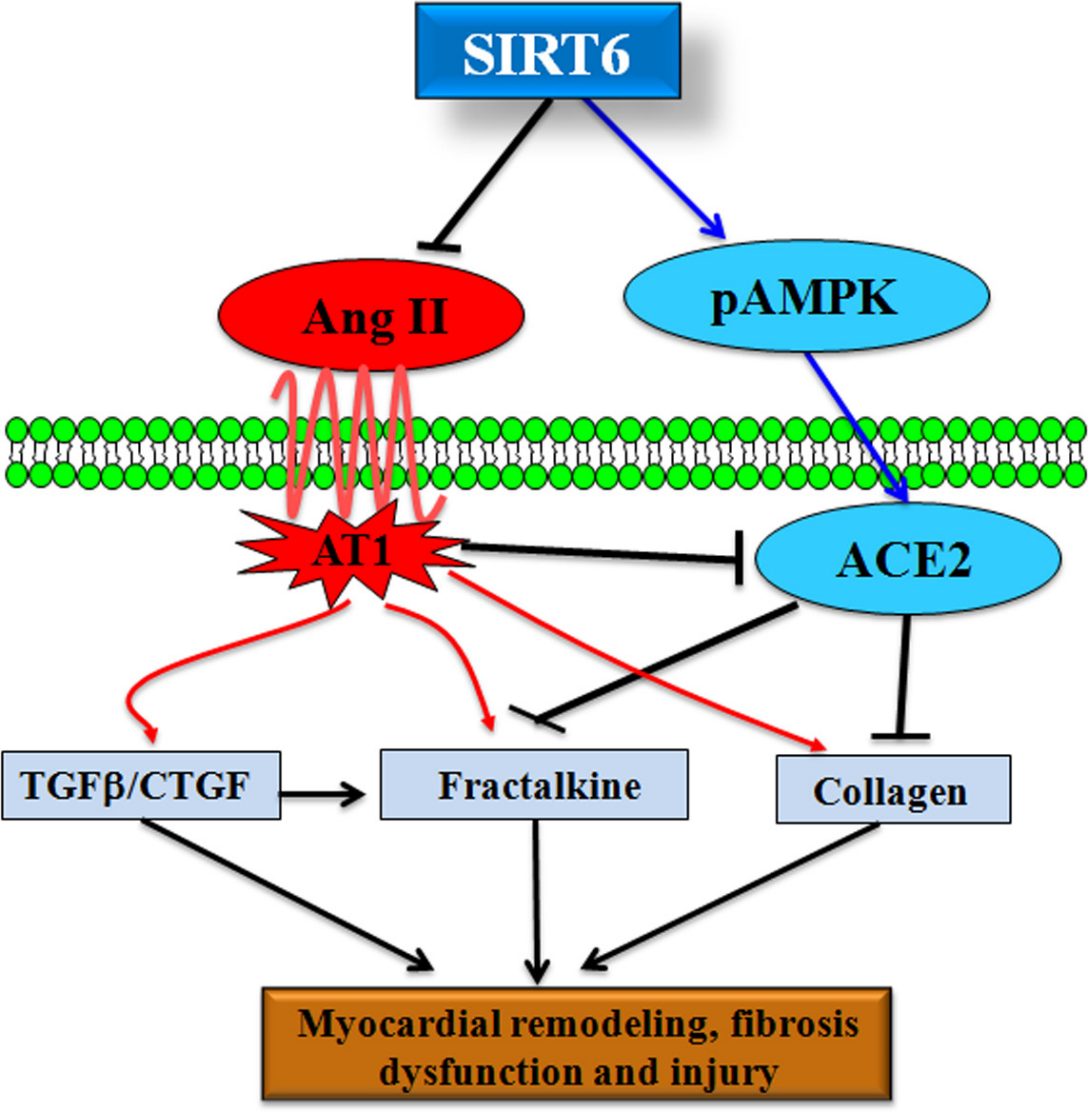
SIRT6 is a negative regulator of Ang II-mediated myocardial remodeling, fibrosis and injury SIRT6 serves as a key regulator of Ang II-mediated hypertension, pathological hypertrophy, myocardial fibrosis and injury in hypertensive rats. Both Ang II infusion and ACE2 deficiency lead to reduction of SIRT6 levels, greater myocardial hypertrophy and impaired heart function. Administration of pAAV-SIRT6 upregulates cardiac levels of p-AMPK and ACE2 and prevents the activation of CTGF-FKN signaling, contributing to the amelioration of Ang II-mediated myocardial hypertrophy, fibrosis, dysfunction and ultrastructure injury in hypertensive rats.

We finally examined the effects of SIRT6 treatment on myocardial CTGF-FKN signaling, fibrosis and collagen production by Picrosirius red staining and Western blotting analyses. Compared with WT control rats, cardiac levels of proinflammatory chemokine FKN (Figures 6A, 6D) and profibrotic factors CTGF (Figure 6C), transforming growth factor-β1 (TGF-β1) (Figure 8C), collagen I (Figures 6E, 8D) and collagen III (Figures 6F, 8E) were upregulated in pAAV-GFP-treated hypertensive rats. These changes were linked with increased myocardial fibrosis (Figures 8A, 8B) and ultrastructural injury (Figure 9), characterized with disruption or dissolution of myocardial myofilaments, myofilaments arranged irregularly and loosely and vacuolar degenerational and swollen mitochondrias. More importantly, pAAV-SIRT6 treatment strikingly alleviated Ang II-induced myocardial fibrosis (Figure 8) and ultrastructural injury (Figure 9) in hypertensive rats with activation of phosphorylated AMPK signaling and downregulated levels of fibrosis-related genes CTGF (Figure 6C), FKN (Figure 6D), TGF-b1(Figure 8E), collagen I (Figures 6E, 8F) and collagen III (Figures 6F, 8G). However, there were no changes in cardiac fibronectin 1 (FN1) levels among groups (Figures 6G, 8H). These observations confirmed cardioprotective effect of SIRT6 on myocardial remodeling, fibrosis, cardiac dysfunction and injury through normalization of AMPK-ACE2 and CTGF-FKN signaling (Figure 10).

## DISCUSSION

In this study, we demonstrate a model of cross-talk between SIRT6 and ACE2 signaling in controlling Ang II-mediated pathological hypertrophy, myocardial remodeling, fibrosis, cardiac dysfunction and injury by enhancing p-AMPKα expression and preventing CTGF/FKN signaling. Increasing evidence suggests that sustained activation of CTGF-FKN signaling is associated with the pathogenesis of fibrotic disorders by regulating diverse cellular processes such as proliferation, migration, survival, and collagen deposition [10, 16–18]. CTGF has been revealed to enhance the levels of inflammatory chemokines FKN [19], which is a newly identified membrane-bound chemokine and plays a vital role in the transition from compensated ventricular remodeling hypertrophy to heart failure through the G protein-coupled chemokine receptor CX3CR1 [11]. We have previously demonstrated that loss of ACE2 leads to greater increases in the Ang II-mediated myocardial inflammation, fibrosis and adverse myocardial injury via the activation of the CTGF-FKN and matrix metalloproteinase signaling, and pre-treatment with the CTGF-neutralizing antibody and recombinant ACE2 significantly abrogates Ang II-induced upregulation of superoxide production and FKN levels in cardiofibroblasts [10, 20]. Recent studies have also showed the cross-talk action between the FKN and Ang II signaling in cardiovascular remodeling and fibrosis [11, 16, 21]. SIRT6 binds to nuclear factor-κlight chain enhancer of activated B cells to negatively regulate their target gene transcription, including FKN [22, 23]. In this work, we revealed that Ang II infusion promoted cardiac levels of proinflammatory chemokine FKN. These changes were linked with increases in plasma Ang II levels and myocardial fibrosis and impaired heart function. In contrast, SIRT6 treatment prevented Ang II-mediated hypertension, pathological hypertrophy, myocardial fibrosis, dysfunction and injury by upregulation of ACE2 levels and suppression of the FKN signaling.

The mammalian genome encodes 7 sirtuin isoforms (SIRT 1 to SIRT7) that vary in their tissue specificity, subcellular localization, enzymatic activity, and targets [22, 24–25]. SIRT6 is a chromatin-associated enzyme involved in deacetylating histone 3 at Lys9 (H3K9) and H3K56, thereby regulating gene expression, cellular metabolism, and the inflammatory response [22]. Previous studies demonstrated that SIRT6 expression is downregulated in human failing hearts and mice hearts subjected to transverse aortic constriction (TAC) or chronic infusion of hypertrophic agonists isoproterenol or Ang II, which may play a pivotal role in the etiology of hypertension [6]. In contrast, SIRT6 overexpression has been shown to activate the AMPK [5]. Intriguingly, AMPK activation ablates Ang II-induced endoplasmic reticulum stress and hypertension [26] and results in increased ACE2 expression [15]. ACE2 maps to a defined quantitative trait locus on the X chromosome in three different rat models of hypertension and targeted disruption of ACE2 in mice results in a severe cardiac contractility defect and increased Ang II levels [2, 27]. In chronic heart failure patients, recombinant human ACE2 (rhACE2) effectively metabolized Ang-(1–10) and Ang II into Ang-(1–9) and Ang-(1–7), respectively. Myocardial Ang II levels in explanted human hearts with dilated cardiomyopathy were elevated and Ang II was effectively converted to Ang-(1–7) by rhACE2 [28], indicating ACE2 being an important negative regulator of the RAS and Ang II-mediated hypertension. In this work, we demonstrated that administration of pAAV-SIRT6 partially prevented Ang II-mediated pressor response at post 4-week treatment, but not at post 1-week treatment, in hypertensive rats with upregulated cardiac levels of ACE2 and pAMPKα, indicating roles of AMPK/ACE2 signaling in hypotensive effects of SIRT6. The deacetylase activity of SIRT6 is decreased in Ang II-induced hypertrophic cardiomyocytes and TAC-induced hypertrophic hearts; while overexpression of wild-type SIRT6 but not its catalytically inactive mutant, attenuates AngII-induced cardiomyocyte hypertrophy [7]. Cardiac-specific overexpression of SIRT6 protected mice from pressure overload and agonist isoproterenol-induced hypertrophy [6, 22]. In this work, we revealed that both Ang II infusion and genetic ACE2 deletion reduced SIRT6 levels and developed pathological hypertrophy and impaired cardiac function. These findings indicate that SIRT6 deficiency is associated with the development of cardiac hypertrophy and heart failure.

SIRT6 was reported to blunt the expression of pro inflammatory and profibrotic genes [23]. In this study, we also demonstrated that SIRT6 treatment significantly blocked cardiac expression of proinflammatory chemokine FKN and profibrotic factors such as TGFβ1, CTGF, collagen I and collagen III. For the reason that cardiac hypertrophy and inflammation are associated with myocardial fibrosis and injury, it is conceivable to think that SIRT6 is an antifibrotic and antiinflammatory sirtuin whose upregulation may help to impede the development of fibrosis- and inflammation-related diseases. SIRT6-mutant mice spontaneously developed concentric cardiac hypertrophy with significantly increased HW/BW ratio, reduced left ventricular internal diameter, reduced fractional shortening as well as increased cardiomyocyte size and interstitial fibrosis [6]. In contrast, SIRT6 overexpression has been shown to promote AMP/ATP and then activate the AMPK-forkhead box O3α axis and further initiated the downstream antioxidant-encoding gene expression (manganese superoxide dismutase and catalase), thereby decreasing cellular levels of oxidative stress and mediating cardioprotective roles in the ischemic heart [5]. Intriguingly, SIRT1 is found to regulate ACE2 expression by activation of AMPK signaling. AMPK activation by 5-amino-4-imidazole-carboxamide riboside (AICAR) results in increased cellular ratio of NAD+ to NADH and increased ACE2 expression [15]. SIRT1, in presence of a possible unknown co-factor, binds to the promoter region of ACE2 and this binding is promoted by AICAR. Intriguingly, the AICAR-induced ACE2 expression is inhibited by an inhibitor of SIRT1, providing strong evidence to the SIRT1 mediated transcriptional regulation of ACE2 under conditions of energy stress [15]. Local RAS exists in the cardiovascular tissues, and ACE2, a negative regulator of the RAS, is present in various cell-types including the cardiofibroblasts, cardiomyocytes and coronary microcirculation [2, 9, 29]. ACE2 plays a critical role in the control of cardiac physiology and its altered expression is linked to major pathophysiological changes of the cardiovascular system and fibrosis-related heart disorder [9, 30, 31]. In this study, we found that SIRT6 treatment remarkably potentiated phosphorylated AMPKα levels and subsequently enhanced ACE2 expression, thereby being responsible for attenuation of pathological hypertrophy, cardiac dysfunction, myocardial fibrosis, and injury in hypertensive rats. These observations confirmed cardioprotective effects of SIRT6 on myocardial remodeling, dysfunction and fibrosis through normalization of AMPK-ACE2 signaling.

In summary, both ACE2 deficiency and Ang II infusion block cardiac expression of SIRT6 and enhance the susceptibility to pathological remodeling, dysfunction and injury. Ang II promotes activation of CTGF-FKN pathway and increases myocardial fibrosis, resulting in adverse ultrastructure deterioration. In contrast, treatment with SIRT6 prevents Ang II-mediated pathological hypertrophy, myocardial fibrosis, impaired heart function and ultrastructural injury in hypertensive rats. These results indicate cardiac protective roles of SIRT6 in the prevention of myocardial remodeling, fibrosis, cardiac dysfunction and injury via activation of AMPK-ACE2 signaling and suppression of the CTGF-FKN pathway and support the notion that SIRT6 functions as a partial agonist of ACE2. Targeting SIRT6 and ACE2 has potential therapeutic importance for controlling myocardial remodeling, fibrosis and injury. Future studies are required to more precisely clarify roles of cross-talk between SIRT6 and ACE2 in cardiac injury and related heart diseases.

## MATERIALS AND METHODS

### Experimental animals and protocols

The twelve-week-old male ACE2KO (Ace2^−/y^) rats were provided from H&D Biotechnology Inc (Wuhan, China) and backcrossed into a pure Sprague-Dawley (SD) rat background by the use of transcription activator-like effector nuclease (TALEN) and CRISPR/ Cas9 technologies as before [32]. DNA sequence chromatograms of polymerase chain reaction (PCR) products from WT and ACE2KO rats illustrated the 13 bp deletion in the homozygous sample (Figure 4). The male SD rats were used to the normal control or randomized to either Ang II (200 ng/kg/min) or saline (control) infusion with an osmotic minipump (model 2004, Alzet Corp, Palo Alto, CA) for 4 weeks as before [10, 31]. The recombinant pAAV-human SIRT6 (pAAV-SIRT6) were purchased from Shanghai Genechem Co.,Ltd. (Shanghai, China) by the use of GV388 BamHI/BamHI vector. The hypertensive SD rats were treated by a rat tail-intravenous injection of pAAV-SIRT6 with the dose of 5 × 10^10^ or pAAV-GFP at 1 week prior to 4-week Ang II infusion. Systolic blood pressure levels of rats were measured non-invasively using the tail-cuff method. All experiments were approved and performed in accordance with *the Guide for the Care and Use of Laboratory Animals* published by the US National Institutes of Health (NIH Publication No.85–23, revised 1996) and the Animal Research Ethics Committee at Shanghai Jiao Tong University School of Medicine.

### Echocardiography

Transthoracic echocardiography for rats was performed and analyzed in a blinded manner as described previously using a Vevo 2100 high-resolution imaging system [2, 33]. Rats were anesthetized with 0.75% isoflurane for the duration of the recordings. M-mode images were obtained for measurements of LV end-systolic diameter (LVESD), left ventricle (LV) end-diastolic diameter (LVEDD), and LV posterior wall thickness (LVPWT). Left ventricle ejection fraction (LVEF) and left ventricle fractional shortening (LVFS) were calculated as measures of systolic function as LVEF (%)=(LVEDV-LDESV)/LVEDV×100 and LVFS (%)= (LVEDD–LVESD)/LVEDD)×100, respectively.

### Quantitative real-time PCR

The mRNA levels of transforming growth factor-β1 (TGFβ1), procollagen I-α1, procollagen III-α1, and fibronectin 1 (FN1) were evaluated by quantitative real-time reverse transcription PCR as previously described [33]. Total RNA was extracted from flash-frozen rat hearts using TRIzol reagent (Invitrogen, CA). The cDNA was synthesized by using the PrimeScript RT reagent kit (TAKARA). A SYBR Premix ExTaq II (TAKARA) was used to perform the quantitative real-time PCR in ABI 7900T Real Time System (Applied. Biosystems, CA). The sequences of the primers were as follows: rat TGFβ1: forward 5′-ATGGTGGACCGCAACAAC-3′, reverse 5′-CAAGGTAACGCCAGGAAT-3′; procollagen I-α1: forward 5′-CACCTACAGCACGCTTG-3′, reverse 5′-GGATGGAGGGAGTTTACAC −3′; procollagen III-α1: forward 5′-CCTCCCAGAACATTACATAC-3′, reverse 5′-CAATGTCATAGGGTGCGAT-3′; FN1: forward 5′- GTGAAGAACGAGGAGGATGTG-3′, reverse 5′-GTGA TGGCGGATGATGTAGC-3′, and GAPDH: forward 5′-C AAGGAGTAAGAAACCCTGGAC-3′, reverse 5′-CTCC TGTTGTTATGGGGTCT-3′. All samples were run in triplicates. GAPDH was used as an endogenous control.

### Western blot analysis

Western blotting analysis was used to measure protein levels of rats hearts as described previously [30, 33]. Protein concentration of the whole tissue lysates was measured using BCA protein assay (BioRad). The whole tissue lysates were subjected to SDS-PAGE and transferred to immobilon^@^P PVDF membranes (Life science) by electro-blotting. The primary antibody against SIRT6 (40 kD), ACE2 (90 kD), p-AMPKα (62 kD), AMPKα (62 kD), CTGF (38 kD), Collage I (150 kD), Collage III (70 kD), FN1 (220 kD) and α-tubulin (55 kD) were obtained from Cell Signaling Technology (Beverly, MA), Abcam Inc. (Cambridge, MA) and Santa Cruz Biotechnology (Santa Cruz, CA), respectively. Aim proteins were detected by enhanced chemiluminescence (GE) and quantified with Image Quant LAS 4000 (GE Healthcare).

### WGA, picro-sirius red staining and confocal laser scanning analyses

Wheat Germ Agglutinin (WGA), H&E and Picro-sirius red (PSR) staining were carried out according to manufacturer's instructions as previously described [14, 20]. PSR staining of heart section was used to assess for myocardial interstitial fibrosis. Confocal laser scanning microscopy was used to test the colocalization SIRT6 of with ACE2 protein in rat hearts by using multi-fluorescent method. Species-appropriate secondary antibodies were conjugated to different fluorophores containing CY3 (Red) and fluorescein isothiocyanate (FITC; Green) in separate channels. Upon nuclear stain and mount in antifade medium containing 4′,6′- diamidino-2-phenylindole (DAPI, blue), immunofluorescence images were acquired using a confocal laser scanning microscope (TCS SP2; Leica Microsystems AG, Wetzlar, Germany).

### Transmission electron microscope array

For transmission electron microscope (TEM) analysis, samples of heart tissues were immediately cut into small pieces and immersed in 2.5% glutaraldehyde as described previously [10, 20]. The myocardial ultrastructure of rat was observed on a conventional scanning transmission electron microscope (Philips, CM120, Amsterdam, Holland).

### Statistical analysis

Values are represented as mean±SEM. All statistical analyses were performed with SPSS software (Version 16.0; Chicago, IL) either by Student's t test or by ANOVA followed by the Student-Newman-Keuls test for multiple-comparison testing as appropriate. A value of *P* < 0.05 was considered to indicate statistically significance.
